# Detecting hallucinations in large language models using semantic entropy

**DOI:** 10.1038/s41586-024-07421-0

**Published:** 2024-06-19

**Authors:** Sebastian Farquhar, Jannik Kossen, Lorenz Kuhn, Yarin Gal

**Affiliations:** OATML, Department of Computer Science, University of Oxford, Oxford, UK

**Keywords:** Computer science, Information technology

## Abstract

Large language model (LLM) systems, such as ChatGPT^1^ or Gemini^2^, can show impressive reasoning and question-answering capabilities but often ‘hallucinate’ false outputs and unsubstantiated answers^3,4^. Answering unreliably or without the necessary information prevents adoption in diverse fields, with problems including fabrication of legal precedents^5^ or untrue facts in news articles^6^ and even posing a risk to human life in medical domains such as radiology^7^. Encouraging truthfulness through supervision or reinforcement has been only partially successful^8^. Researchers need a general method for detecting hallucinations in LLMs that works even with new and unseen questions to which humans might not know the answer. Here we develop new methods grounded in statistics, proposing entropy-based uncertainty estimators for LLMs to detect a subset of hallucinations—confabulations—which are arbitrary and incorrect generations. Our method addresses the fact that one idea can be expressed in many ways by computing uncertainty at the level of meaning rather than specific sequences of words. Our method works across datasets and tasks without a priori knowledge of the task, requires no task-specific data and robustly generalizes to new tasks not seen before. By detecting when a prompt is likely to produce a confabulation, our method helps users understand when they must take extra care with LLMs and opens up new possibilities for using LLMs that are otherwise prevented by their unreliability.

## Abstract (web summary)

Hallucinations (confabulations) in large language model systems can be tackled by measuring uncertainty about the meanings of generated responses rather than the text itself to improve question-answering accuracy.

## Main

‘Hallucinations’ are a critical problem^9^ for natural language generation systems using large language models (LLMs), such as ChatGPT^1^ or Gemini^2^, because users cannot trust that any given output is correct.

Hallucinations are often defined as LLMs generating “content that is nonsensical or unfaithful to the provided source content”^9–11^ but they have come to include a vast array of failures of faithfulness and factuality. We focus on a subset of hallucinations which we call ‘confabulations’^12^ for which LLMs fluently make claims that are both wrong and arbitrary—by which we mean that the answer is sensitive to irrelevant details such as random seed. For example, when asked a medical question “What is the target of Sotorasib?” an LLM confabulates by sometimes answering KRASG12 ‘C’ (correct) and other times KRASG12 ‘D’ (incorrect) despite identical instructions. We distinguish this from cases in which a similar ‘symptom’ is caused by the following different mechanisms: when LLMs are consistently wrong as a result of being trained on erroneous data such as common misconceptions^13^; when the LLM ‘lies’ in pursuit of a reward^14^; or systematic failures of reasoning or generalization. We believe that combining these distinct mechanisms in the broad category hallucination is unhelpful. Our method makes progress on a portion of the problem of providing scalable oversight^15^ by detecting confabulations that people might otherwise find plausible. However, it does not guarantee factuality because it does not help when LLM outputs are systematically bad. Nevertheless, we significantly improve question-answering accuracy for state-of-the-art LLMs, revealing that confabulations are a great source of error at present.

We show how to detect confabulations by developing a quantitative measure of when an input is likely to cause an LLM to generate arbitrary and ungrounded answers. Detecting confabulations allows systems built on LLMs to avoid answering questions likely to cause confabulations, to make users aware of the unreliability of answers to a question or to supplement the LLM with more grounded search or retrieval. This is essential for the critical emerging field of free-form generation in which naive approaches, suited to closed vocabulary and multiple choice, fail. Past work on uncertainty for LLMs has focused on simpler settings, such as classifiers^16,17^ and regressors^18,19^, whereas the most exciting applications of LLMs relate to free-form generations.

The term hallucination in the context of machine learning originally comes from filling in ungrounded details, either as a deliberate strategy^20^ or as a reliability problem^4^. The appropriateness of the metaphor has been questioned as promoting undue anthropomorphism^21^. Although we agree that metaphor must be used carefully with LLMs^22^, the widespread adoption of the term hallucination reflects the fact that it points to an important phenomenon. This work represents a step towards making that phenomenon more precise.

To detect confabulations, we use probabilistic tools to define and then measure the ‘semantic’ entropy of the generations of an LLM—an entropy that is computed over meanings of sentences. High entropy corresponds to high uncertainty^23–25^—so semantic entropy is one way to estimate semantic uncertainties. Semantic uncertainty, the broader category of measures we introduce, could be operationalized with other measures of uncertainty, such as mutual information, instead. Entropy in free-form generation is normally hard to measure because answers might mean the same thing (be semantically equivalent) despite being expressed differently (being syntactically or lexically distinct). This causes naive estimates of entropy or other lexical variation scores^26^ to be misleadingly high when the same correct answer might be written in many ways without changing its meaning.

**Fig. 1:**
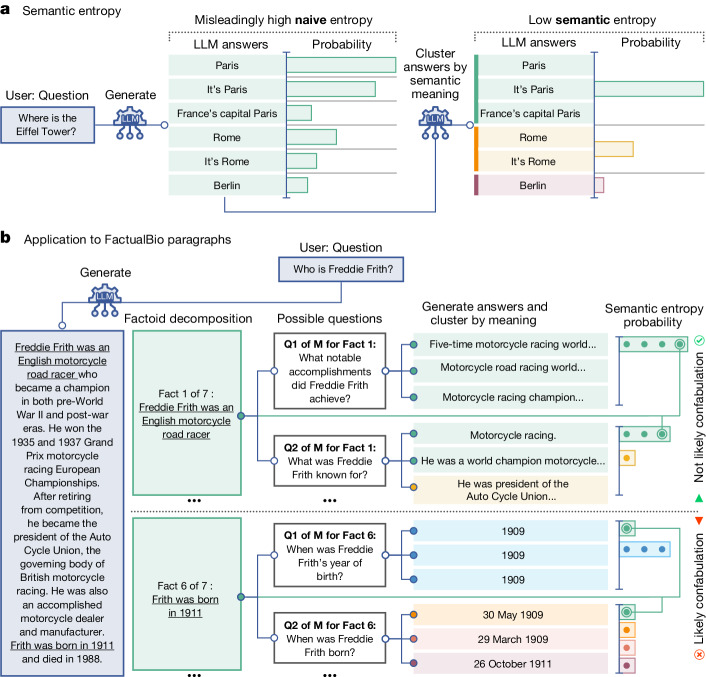
Overview of semantic entropy and confabulation detection. **a**, Naive entropy-based uncertainty measures variation in the exact answers, treating ‘Paris’, ‘It’s Paris’ and ‘France’s capital Paris’ as different. But this is unsuitable for language tasks for which sometimes different answers mean the same things. Our semantic entropy clusters answers which share meanings before computing the entropy. A low semantic entropy shows that the LLM is confident about the meaning. **b**, Semantic entropy can also detect confabulations in longer passages. We automatically decompose a long generated answer into factoids. For each factoid, an LLM generates questions to which that factoid might have been the answer. The original LLM then samples *M* possible answers to these questions. Finally, we compute the semantic entropy over the answers to each specific question, including the original factoid. Confabulations are indicated by high average semantic entropy for questions associated with that factoid. Here, semantic entropy classifies Fact 1 as probably not a confabulation because generations often mean the same thing, despite very different wordings, which a naive entropy would have missed.

By contrast, our semantic entropy moves towards estimating the entropy of the distribution of meanings of free-form answers to questions, insofar as that is possible, rather than the distribution over the ‘tokens’ (words or word-pieces) which LLMs natively represent. This can be seen as a kind of semantic consistency check^27^ for random seed variation. An overview of our approach is provided in Fig. 1 and a worked example in Supplementary Table 1.

Intuitively, our method works by sampling several possible answers to each question and clustering them algorithmically into answers that have similar meanings, which we determine on the basis of whether answers in the same cluster entail each other bidirectionally^28^. That is, if sentence A entails that sentence B is true and vice versa, then we consider them to be in the same semantic cluster. We measure entailment using both general-purpose LLMs and natural language inference (NLI) tools developed specifically for detecting entailment for which we show direct evaluations in Supplementary Tables 2 and 3 and Supplementary Fig. 1. Textual entailment has previously been shown to correlate with faithfulness^10^ in the context of factual consistency^29^ as well as being used to measure factuality in abstractive summarization^30^, especially when applied at the right granularity^31^.

Semantic entropy detects confabulations in free-form text generation across a range of language models and domains, without previous domain knowledge. Our evaluations cover question answering in trivia knowledge (TriviaQA^32^), general knowledge (SQuAD 1.1; ref. ^33^), life sciences (BioASQ^34^) and open-domain natural questions (NQ-Open^35^) derived from actual queries to Google Search^36^. In addition, semantic entropy detects confabulations in mathematical word problems (SVAMP^37^) and in a biography-generation dataset, FactualBio, accompanying this paper.

Our results for TriviaQA, SQuAD, BioASQ, NQ-Open and SVAMP are all evaluated context-free and involve sentence-length answers (96 ± 70 characters, mean ± s.d.) and use LLaMA 2 Chat (7B, 13B and 70B parameters)^38^, Falcon Instruct (7B and 40B)^39^ and Mistral Instruct (7B)^40^. In the Supplementary Information, we further consider short-phrase-length answers. Results for FactualBio (442 ± 122 characters) use GPT-4 (ref. ^1^). At the time of writing, GPT-4 (ref. ^1^) did not expose output probabilities^41^ or hidden states, although it does now. As a result, we propose a discrete approximation of our estimator for semantic entropy which allows us to run experiments without access to output probabilities, which we use for all GPT-4 results in this paper and which performs similarly well.

Our confabulation detection with semantic entropy is more robust to user inputs from previously unseen domains than methods which aim to ‘learn’ how to detect confabulations from a set of example demonstrations. Our method is unsupervised, meaning that we do not need labelled examples of confabulations. By contrast, supervised methods detect confabulations by learning patterns behind examples of confabulations, assuming that future questions preserve these patterns. But this assumption is often untrue in new situations or with confabulations that human overseers are unable to identify (compare Fig. 17 of ref. ^24^). As a strong supervised baseline, we compare to an embedding regression method inspired by ref. ^24^ which trains a logistic regression classifier to predict whether the model correctly answered a question on the basis of the final ‘embedding’ (hidden state) of the LLM. We also use the *P*(True) method^24^ which looks at the probability with which an LLM predicts that the next token is ‘True’ when few-shot prompted to compare a main answer with ‘brainstormed’ alternatives.

Confabulations contribute substantially to incorrect answers given by language models. We show that semantic entropy can be used to predict many incorrect model answers and to improve question-answering accuracy by refusing to answer those questions the model is uncertain about. Corresponding to these two uses, we evaluate two main metrics. First, the widely used area under the receiver operating characteristic (AUROC) curve for the binary event that a given answer is incorrect. This measure captures both precision and recall and ranges from 0 to 1, with 1 representing a perfect classifier and 0.5 representing an un-informative classifier. We also show a new measure, the area under the ‘rejection accuracy’ curve (AURAC). This studies the case in which the confabulation detection score is used to refuse to answer the questions judged most likely to cause confabulations. Rejection accuracy is the accuracy of the answers of the model on the remaining questions and the area under this curve is a summary statistic over many thresholds (representative threshold accuracies are provided in Supplementary Material). The AURAC captures the accuracy improvement which users would experience if semantic entropy was used to filter out questions causing the highest entropy.

## Detecting confabulations in QA and math

**Fig. 2:**
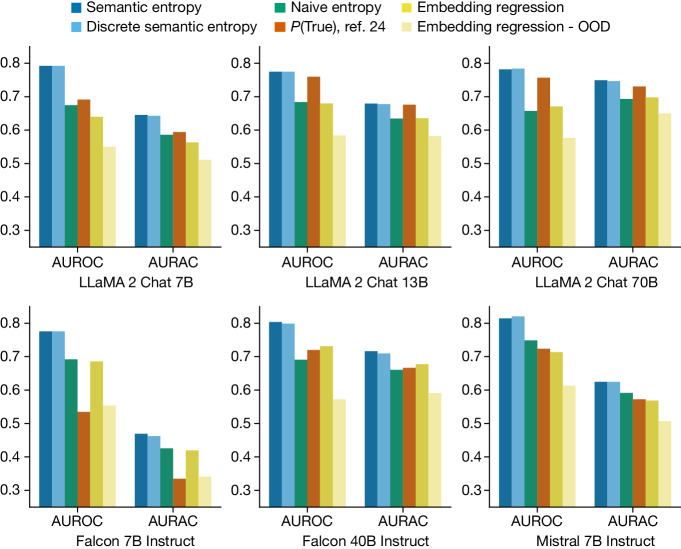
Detecting confabulations in sentence-length generations. Semantic entropy outperforms leading baselines and naive entropy. AUROC (scored on the *y*-axes) measures how well methods predict LLM mistakes, which correlate with confabulations. AURAC (likewise scored on the *y*-axes) measures the performance improvement of a system that refuses to answer questions which are judged likely to cause confabulations. Results are an average over five datasets, with individual metrics provided in the Supplementary Information.

In Fig. 2, we show that both semantic entropy and its discrete approximation outperform our best baselines for sentence-length generations. These results are averaged across datasets and provide the actual scores on the held-out evaluation dataset. We report the raw average score across held-out evaluation datasets without standard error because the distributional characteristics are more a property of the models and datasets selected than the method. Consistency of relative results across different datasets is a stronger indicator of variation in this case.

Semantic entropy greatly outperforms the naive estimation of uncertainty using entropy: computing the entropy of the length-normalized joint probability of the token sequences. Naive entropy estimation ignores the fact that token probabilities also express the uncertainty of the model over phrasings that do not change the meaning of an output.

Our methods also outperform the supervised embedding regression method both in- and out-of-distribution. In pale-yellow bars we show that embedding regression performance deteriorates when its training data do not match the deployment distribution—which mirrors the common real-world case in which there is a distribution shift between training and deployment^42^—the plotted value is the average metric for embedding regression trained on one of the four ‘off-distribution’ datasets for that evaluation. This is critical because reliable uncertainty is most important when the data distribution shifts. Semantic entropy also outperforms *P*(True) which is supervised ‘in-context’; that is, it is adapted to the deployment task with a few training examples provided in the LLM prompt itself. The discrete variant of semantic entropy performs similarly to our standard estimator, despite not requiring exact output probabilities.

Averaged across the 30 combinations of tasks and models we study, semantic entropy achieves the best AUROC value of 0.790 whereas naive entropy (0.691), *P*(True) (0.698) and the embedding regression baseline (0.687) lag behind it. Semantic entropy performs well consistently, with stable performance (between 0.78 and 0.81 AUROC) across the different model families (LLaMA, Falcon and Mistral) and scales (from 7B to 70B parameters) which we study (we report summary statistics for each dataset and model as before). Although semantic entropy outperforms the baselines across all model sizes, *P*(True) seems to improve with model size, suggesting that it might become more competitive for very capable honest models in settings that the model understands well (which are, however, not the most important cases to have good uncertainty). We use ten generations to compute entropy, selected using analysis in Supplementary Fig. 2. Further results for short-phrase generations are described in Supplementary Figs. 7–10.

The results in Fig. 2 offer a lower bound on the effectiveness of semantic entropy at detecting confabulations. These evaluations determine whether semantic entropy and baseline methods can detect when the answers of the model are incorrect (which we validate against human correctness evaluations in Supplementary Table 4). In addition to errors from confabulations (arbitrary incorrectness), this also includes other types of mistakes for which semantic entropy is not suited, such as consistent errors learned from the training data. The fact that methods such as embedding regression are able to spot other kinds of errors, not just confabulations, but still are outperformed by semantic entropy, suggests that confabulations are a principal category of errors for actual generations.

Examples of questions and answers from TriviaQA, SQuAD and BioASQ, for LLaMA 2 Chat 70B, are shown in Table 1. These illustrate how only semantic entropy detects when the meaning is constant but the form varies (the first row of the table) whereas semantic entropy and naive entropy both correctly predict the presence of confabulations when the form and meaning vary together (second row) and predict the absence of confabulations when the form and meaning are both constant across several resampled generations (third row). In the final row, we give an example in which semantic entropy is erroneously high as a result of overly sensitive semantic clustering relative to the reference answer. Our clustering method distinguishes the answers which provide a precise date from those which only provide a year. For some contexts that would have been correct but in this context the distinction between the specific day and the year is probably irrelevant. This highlights the importance of context and judgement in clustering, especially in subtle cases, as well as the shortcomings of evaluating against fixed reference answers which do not capture the open-ended flexibility of conversational deployments of LLMs.

**Table 1:** Semantic entropy applied to examples

Question	Model answer	Reference answer	Generations for entropy	Prediction	
				Semantic entropy	Naive entropy
Refineries, process chemical, power generation, mills and manufacturing plants are under what sector of construction?	Refineries, process chemical, power generation, mills and manufacturing plants are under the industrial sector of construction.	Industrial or heavy civil or heavy engineering.	• All the above are under the industrial sector of construction.	Not confabulation	Confabulation
			• The refineries, process chemical, power generation, mills and manufacturing plants are under the industrial sector of construction.		
			• These are all under the heavy industrial sector of construction.		
What is the function of the stard10 protein?	The stard10 protein functions as a negative regulator of the mTOR pathway by inhibiting the activity of the mTORC1 and mTORC2 complexes.	STARD10 is a lipid transfer protein.	• The stard10 protein plays a crucial role in regulating the dynamics of the actin cytoskeleton and cell adhesion during cell migration and cell division.	Confabulation	Confabulation
			• The stard10 protein functions as a negative regulator of the meiotic recombination process, specifically by inhibiting the activity of the Dmc1 recombinase enzyme.		
			• The stard10 protein functions as a regulator of lipid metabolism, promoting the breakdown of lipids in the liver and inhibiting their synthesis in the liver and adipose tissue.		
Of which European country is ‘fado’ the national music?	Portugal.	Portugal.	• ‘Fado’ is the national music of Portugal.	Not confabulation	Not confabulation
			• ‘Fado’ is the national music of Portugal.		
			• Fado is the national music of Portugal.		
When was BSkyB’s digital service launched?	1 October 1998.	BSkyB’s digital service was launched in 1998.	• BSkyB’s digital service was launched in 1998.	Confabulation	Not confabulation
			• BSkyB’s digital service was launched on 1 October 1998.		
			• BSkyB’s digital service was launched on 1 October 1998.		

The first row of Table 1 demonstrates a case in which semantic entropy correctly predicts that an answer is not a confabulation if naive entropy would incorrectly predict a confabulation. All of the generations from the model mean the same thing as each other so they are clustered together despite using different phrasings. The second row provides an example in which semantic entropy and naive entropy would both correctly predict a confabulation, in which each generation is both lexically distinct and also means something different. The third row is an example in which semantic entropy and naive entropy would both correctly predict no confabulation because the multiple generations are almost lexically identical. The fourth row gives an example in which semantic entropy might fail but naive entropy might succeed. In our experiment, semantic entropy clustered the answers into those which provided a specific date and those which gave only a year and treated the model as ‘uncertain’. This highlights the importance of context in semantic clustering. The examples come from LLaMA 2 Chat 70B generations for SQuAD, BioASQ and TriviaQA.

## Detecting confabulations in biographies

Semantic entropy is most natural for sentences that express a single proposition but the idea of semantic equivalence is trickier to apply to longer passages which express many propositions which might only agree partially^43^. Nevertheless, we can use semantic entropy to detect confabulations in longer generations, such as entire paragraphs of text. To show this, we develop a dataset of biographical generations from GPT-4 (v.0613) for 21 individuals notable enough to have their own Wikipedia page but without extensive online biographies. From each biography generated by GPT-4, we automatically extract propositional factual claims about the individual (150 factual claims in total), which we manually label as true or false.

Applying semantic entropy to this problem is challenging. Naively, one might simply regenerate each sentence (conditioned on the text so far) and then compute semantic entropy over these regenerations. However, the resampled sentences often target different aspects of the biography: for example, one time describing family and the next time profession. This is analogous to the original problem semantic entropy was designed to resolve: the model is uncertain about the right ordering of facts, not about the facts themselves. To address this, we break down the entire paragraph into factual claims and reconstruct questions which might have been answered by those claims. Only then do we apply semantic entropy (Fig. 1) by generating three new answers to each question (selected with analysis in Supplementary Figs. 3 and 4) and computing the semantic entropy over those generations plus the original factual claim. We aggregate these by averaging the semantic entropy over all the questions to get an uncertainty score for each proposition, which we use to detect confabulations. Unaggregated results are shown in Supplementary Figs. 5 and 6.

As GPT-4 did not allow access to the probability of the generation at the time of writing, we use a discrete variant of semantic entropy which makes the further approximation that we can infer a discrete empirical distribution over semantic meaning clusters from only the generations (Methods). This allows us to compute semantic entropy using only the black-box outputs of an LLM. However, we were unable to compute the naive entropy baseline, the standard semantic entropy estimator or the embedding regression baseline for GPT-4 without output probabilities and embeddings.

**Fig. 3:**
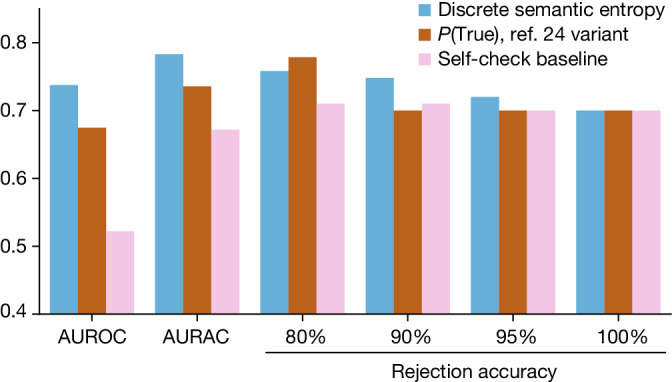
Detecting GPT-4 confabulations in paragraph-length biographies. The discrete variant of our semantic entropy estimator outperforms baselines both when measured by AUROC and AURAC metrics (scored on the *y*-axis). The AUROC and AURAC are substantially higher than for both baselines. At above 80% of questions being answered, semantic entropy has the highest accuracy. Only when the top 20% of answers judged most likely to be confabulations are rejected does the answer accuracy on the remainder for the *P*(True) baseline exceed semantic entropy.

In Fig. 3 we show that the discrete variant of semantic entropy effectively detects confabulations on this dataset. Its AUROC and AURAC are higher than either a simple ‘self-check’ baseline—which just asks the LLM whether the factoid is likely to be true—or a variant of *P*(True) which has been adapted to work for the paragraph-length setting. Discrete semantic entropy has better rejection accuracy performance until 20% of the questions have been rejected at which point *P*(True) has a narrow edge. This indicates that the questions predicted to cause confabulations are indeed more likely to be wrong.

## Discussion

Our probabilistic approach, accounting for semantic equivalence, detects an important class of hallucinations: those that are caused by a lack of LLM knowledge. These are a substantial portion of the failures at present and will continue even as models grow in capabilities because situations and cases that humans cannot reliably supervise will persist. Confabulations are a particularly noteworthy failure mode for question answering but appear in other domains too. Semantic entropy needs no previous domain knowledge and we expect that algorithmic adaptations to other problems will allow similar advances in, for example, abstractive summarization. In addition, extensions to alternative input variations such as rephrasing or counterfactual scenarios would allow a similar method to act as a form of cross-examination^44^ for scalable oversight through debate^45^.

The success of semantic entropy at detecting errors suggests that LLMs are even better at “knowing what they don’t know” than was argued by ref. ^24^—they just don’t know they know what they don’t know. Our method explicitly does not directly address situations in which LLMs are confidently wrong because they have been trained with objectives that systematically produce dangerous behaviour, cause systematic reasoning errors or are systematically misleading the user. We believe that these represent different underlying mechanisms—despite similar ‘symptoms’—and need to be handled separately.

One exciting aspect of our approach is the way it makes use of classical probabilistic machine learning methods and adapts them to the unique properties of modern LLMs and free-form language generation. We hope to inspire a fruitful exchange of well-studied methods and emerging new problems by highlighting the importance of meaning when addressing language-based machine learning problems.

## Methods

Semantic entropy as a strategy for overcoming confabulation builds on probabilistic tools for uncertainty estimation. It can be applied directly to any LLM or similar foundation model without requiring any modifications to the architecture. Our ‘discrete’ variant of semantic uncertainty can be applied even when the predicted probabilities for the generations are not available, for example, because access to the internals of the model is limited.

In this section we introduce background on probabilistic methods and uncertainty in machine learning, discuss how it applies to language models and then discuss our contribution, semantic entropy, in detail.

### Background

#### Uncertainty and machine learning

We aim to detect confabulations in LLMs, using the principle that the model will be uncertain about generations for which its output is going to be arbitrary.

One measure of uncertainty is the predictive entropy of the output distribution, which measures the information one has about the output given the input^25^. The predictive entropy (PE) for an input sentence **x** is the conditional entropy (*H*) of the output random variable *Y* with realization *y* given **x**,

1 $${\rm{PE}}({\bf{x}})=H(Y| {\bf{x}})=-\sum _{y}P(\,y| {\bf{x}})\mathrm{ln}P(\,y| {\bf{x}}).$$

A low predictive entropy indicates an output distribution which is heavily concentrated whereas a high predictive entropy indicates that many possible outputs are similarly likely.

#### Aleatoric and epistemic uncertainty

We do not distinguish between aleatoric and epistemic uncertainty in our analysis. Researchers sometimes separate aleatoric uncertainty (uncertainty in the underlying data distribution) from epistemic uncertainty (caused by having only limited information)^46^. Further advances in uncertainty estimation which separate these kinds of uncertainty would enhance the potential for our semantic uncertainty approach by allowing extensions beyond entropy.

#### Joint probabilities of sequences of tokens

Generative LLMs produce strings of text by selecting tokens in sequence. Each token is a wordpiece that often represents three or four characters (though especially common sequences and important words such as numbers typically get their own token). To compute entropies, we need access to the probabilities the LLM assigns to the generated sequence of tokens. The probability of the entire sequence, **s**, conditioned on the context, **x**, is the product of the conditional probabilities of new tokens given past tokens, whose resulting log-probability is $\log P({\bf{s}}| {\boldsymbol{x}})={\sum }_{i}\log P({s}_{i}| {{\bf{s}}}_{ < i},{\boldsymbol{x}})$, where *s*_*i*_ is the *i*th output token and **s**_<*i*_ denotes the set of previous tokens.

#### Length normalization

When comparing the log-probabilities of generated sequences, we use ‘length normalization’, that is, we use an arithmetic mean log-probability, $\frac{1}{N}{\sum }_{i}^{N}\log P({s}_{i}| {{\bf{s}}}_{ < i},{\boldsymbol{x}})$, instead of the sum. In expectation, longer sequences have lower joint likelihoods because of the conditional independence of the token probabilities^47^. The joint likelihood of a sequence of length *N* shrinks exponentially in *N*. Its negative log-probability therefore grows linearly in *N*, so longer sentences tend to contribute more to entropy. We therefore interpret length-normalizing the log-probabilities when estimating the entropy as asserting that the expected uncertainty of generations is independent of sentence length. Length normalization has some empirical success^48^, including in our own preliminary experiments, but little theoretical justification in the literature.

### Principles of semantic uncertainty

If we naively calculate the predictive entropy directly from the probabilities of the generated sequence of tokens, we conflate the uncertainty of the model over the meaning of its answer with the uncertainty over the exact tokens used to express that meaning. For example, even if the model is confident in the meaning of a generation, there are still usually many different ways for phrasing that generation without changing its meaning. For the purposes of detecting confabulations, the uncertainty of the LLM over meanings is more important than the uncertainty over the exact tokens used to express those meanings.

Our semantic uncertainty method therefore seeks to estimate only the uncertainty the LLM has over the meaning of its generation, not the choice of words. To do this, we introduce an algorithm that clusters model generations by meaning and subsequently calculates semantic uncertainty. At a high level this involves three steps:

1. Generation: sample output sequences of tokens from the predictive distribution of a LLM given a context **x**.
2. Clustering: cluster sequences by their meaning using our clustering algorithm based on bidirectional entailment.
3. Entropy estimation: estimate semantic entropy by summing probabilities of sequences that share a meaning following equation (2) and compute their entropy.

#### Generating a set of answers from the model

Given some context **x** as input to the LLM, we sample *M* sequences, {**s**^(1)^, …, **s**^(*M*)^} and record their token probabilities, {*P*(**s**^(1)^∣**x**), …, *P*(**s**^(*M*)^∣**x**)}. We sample all our generations from a single model, varying only the random seed used for sampling from the token probabilities. We do not observe the method to be particularly sensitive to details of the sampling scheme. In our implementation, we sample at temperature 1 using nucleus sampling (*P* = 0.9) (ref. ^49^) and top-*K* sampling (*K* = 50) (ref. ^50^). We also sample a single generation at low temperature (0.1) as an estimate of the ‘best generation’ of the model to the context, which we use to assess the accuracy of the model. (A lower sampling temperature increases the probability of sampling the most likely tokens).

#### Clustering by semantic equivalence

To estimate semantic entropy we need to cluster generated outputs from the model into groups of outputs that mean the same thing as each other.

This can be described using ‘semantic equivalence’ which is the relation that holds between two sentences when they mean the same thing. We can formalize semantic equivalence mathematically. Let the space of tokens in a language be ${\mathcal{T}}$. The space of all possible sequences of tokens of length *N* is then ${{\mathcal{S}}}_{N}\equiv {{\mathcal{T}}}^{N}$. Note that *N* can be made arbitrarily large to accommodate whatever size of sentence one can imagine and one of the tokens can be a ‘padding’ token which occurs with certainty for each token after the end-of-sequence token. For some sentence ${\bf{s}}\in {{\mathcal{S}}}_{N}$, composed of a sequence of tokens, ${s}_{i}\in {\mathcal{T}}$, there is an associated meaning. Theories of meaning are contested^51^. However, for specific models and deployment contexts many considerations can be set aside. Care should be taken comparing very different models and contexts.

Let us introduce a semantic equivalence relation, *E*( ⋅ , ⋅ ), which holds for any two sentences that mean the same thing—we will operationalize this presently. Recall that an equivalence relation is any reflexive, symmetric and transitive relation and that any equivalence relation on a set corresponds to a set of equivalence classes. Each semantic equivalence class captures outputs that can be considered to express the same meaning. That is, for the space of semantic equivalence classes ${\mathcal{C}}$ the sentences in the set $c\in {\mathcal{C}}$ can be regarded in many settings as expressing a similar meaning such that $\forall {\bf{s}},{{\bf{s}}}^{{\prime} }\in c:E({\bf{s}},{{\bf{s}}}^{{\prime} })$. So we can build up these classes of semantically equivalent sentences by checking if new sentences share a meaning with any sentences we have already clustered and, if so, adding them into that class.

We operationalize *E*( ⋅ , ⋅ ) using the idea of bidirectional entailment, which has a long history in linguistics^52^ and natural language processing^28,53,54^. A sequence, **s**, means the same thing as a second sequence, **s**′, only if the sequences entail (that is, logically imply) each other. For example, ‘The capital of France is Paris’ entails ‘Paris is the capital of France’ and vice versa because they mean the same thing. (See later for a discussion of soft equivalence and cases in which bidirectional entailment does not guarantee equivalent meanings).

Importantly, we require that the sequences mean the same thing with respect to the context—key meaning is sometimes contained in the context. For example, ‘Paris’ does not entail ‘The capital of France is Paris’ because ‘Paris’ is not a declarative sentence without context. But in the context of the question ‘What is the capital of France?’, the one-word answer does entail the longer answer.

Detecting entailment has been the object of study of a great deal of research in NLI^55^. We rely on language models to predict entailment, such as DeBERTa-Large-MNLI^56^, which has been trained to predict entailment, or general-purpose LLMs such as GPT-3.5 (ref. ^57^), which can predict entailment given suitable prompts.

**Extended Data Fig. 1:**
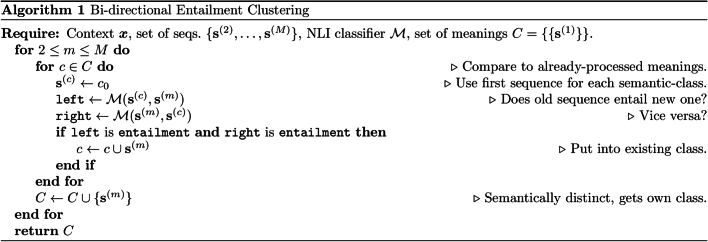
Algorithm outline for bidirectional entailment clustering. Given a set of outputs in response to a context, the bidirectional entailment answer returns a set of sets of outputs which have been classified as sharing a meaning.

We then cluster sentences according to whether they bidirectionally entail each other using the algorithm presented in Extended Data Fig. 1. Note that, to check if a sequence should be added to an existing cluster, it is sufficient to check if the sequence bidirectionally entails any of the existing sequences in that cluster (we arbitrarily pick the first one), given the transitivity of semantic equivalence. If a sequence does not share meaning with any existing cluster, we assign it its own cluster.

#### Computing the semantic entropy

Having determined the classes of generated sequences that mean the same thing, we can estimate the likelihood that a sequence generated by the LLM belongs to a given class by computing the sum of the probabilities of all the possible sequences of tokens which can be considered to express the same meaning as

2 $$P(c| {\boldsymbol{x}})=\sum _{{\bf{s}}\in c}P({\bf{s}}| {\boldsymbol{x}})=\sum _{{\bf{s}}\in c}\prod _{i}P({s}_{i}| {{\bf{s}}}_{ < i},{\boldsymbol{x}}).$$

Formally, this treats the output as a random variable whose event-space is the space of all possible meaning-classes, *C*, a sub-*σ*-algebra of the standard event-space *S*. We can then estimate the semantic entropy (SE) as the entropy over the meaning-distribution,

3 $${\rm{SE}}(x)=-\sum _{c}P(c| {\boldsymbol{x}})\log P(c| {\boldsymbol{x}})$$

4 $$=-\sum _{c}\left(\left[\sum _{{\bf{s}}\in c}P({\bf{s}}| {\boldsymbol{x}})\right]\log \left[\sum _{{\bf{s}}\in c}P({\bf{s}}| {\boldsymbol{x}})\right]\right).$$

There is a complication which prevents direct computation: we do not have access to every possible meaning-class *c*. Instead, we can only sample *c* from the sequence-generating distribution induced by the model. To handle this, we estimate the expectation in equation (3) using a Rao–Blackwellized Monte Carlo integration over the semantic equivalence classes *C*,

5 $$\begin{array}{r}{\rm{SE}}(x)\approx -\mathop{\sum }\limits_{i=1}^{| C| }P({C}_{i}| {\boldsymbol{x}})\log P({C}_{i}| {\boldsymbol{x}}),\end{array}$$

where $P({C}_{i}| {\boldsymbol{x}})=\frac{P({c}_{i}| {\boldsymbol{x}})}{{\sum }_{c}P(c| {\boldsymbol{x}})}$ estimates a categorical distribution over the cluster meanings, that is, ∑_*i*_*P*(*C*_*i*_∣**x**) = 1. Without this normalization step cluster ‘probabilities’ could exceed one because of length normalization, resulting in degeneracies. Equation (5) is the estimator giving our main method that we refer to as semantic entropy throughout the text.

For scenarios in which the sequence probabilities are not available, we propose a variant of semantic entropy which we call ‘discrete’ semantic entropy. Discrete semantic entropy approximates *P*(*C*_*i*_∣**x**) directly from the number of generations in each cluster, disregarding the token probabilities. That is, we approximate *P*(*C*_*i*_∣**x**) as ${\sum }_{1}^{M}\frac{{I}_{c={C}_{i}}}{M}$, the proportion of all the sampled answers which belong to that cluster. Effectively, this just assumes that each output that was actually generated was equally probable—estimating the underlying distribution as the categorical empirical distribution. In the limit of *M* the estimator converges to equation (5) by the law of large numbers. We find that discrete semantic entropy results in similar performance empirically.

We provide a worked example of the computation of semantic entropy in Supplementary Note 1.

### Detecting confabulations in QA and math

Semantic entropy is designed to detect confabulations, that is, model outputs with arbitrary meaning. In our experiments, we use semantic uncertainty to predict model accuracy, demonstrating that confabulations make up a notable fraction of model mistakes. We further show that semantic uncertainty can be used to improve model accuracy by refusing to answer questions when semantic uncertainty is high. Last, semantic uncertainty can be used to give users a way to know when model generations are probably unreliable.

#### Tasks

We use the datasets BioASQ^34^, SQuAD^33^, TriviaQA^32^, SVAMP^37^ and NQ-Open^35^. BioASQ is a life-sciences question-answering dataset based on the annual challenge of the same name. The specific dataset we use is based on the QA dataset from Task B of the 2023 BioASQ challenge (11B). SQuAD is a reading comprehension dataset whose context passages are drawn from Wikipedia and for which the answers to questions can be found in these passages. We use SQuAD 1.1 which excludes the unanswerable questions added in v.2.0 that are deliberately constructed to induce mistakes so they do not in practice cause confabulations to occur. TriviaQA is a trivia question-answering dataset. SVAMP is a word-problem maths dataset containing elementary-school mathematical reasoning tasks. NQ-Open is a dataset of realistic questions aggregated from Google Search which have been chosen to be answerable without reference to a source text. For each dataset, we use 400 train examples and 400 test examples randomly sampled from the original larger dataset. Note that only some of the methods require training, for example semantic entropy does not use the training data. If the datasets themselves are already split into train and test (or validation) samples, we sample our examples from within the corresponding split.

All these datasets are free-form, rather than multiple choice, because this better captures the opportunities created by LLMs to produce free-form sentences as answers. We refer to this default scenario as our ‘sentence-length’ experiments. In Supplementary Note 7, we also present results for confabulation detection in a ‘short-phrase’ scenario, in which we constrain model answers on these datasets to be as concise as possible.

To make the problems more difficult and induce confabulations, we do not provide the context passages for any of the datasets. When the context passages are provided, the accuracy rate is too high for these datasets for the latest generations of models to meaningfully study confabulations.

#### Models

For sentence-length generations we use: Falcon^39^ Instruct (7B and 40B), LLaMA 2 Chat^38^ (7B, 13B and 70B) and Mistral^40^ Instruct (7B).

#### Baselines

In addition to reporting results for semantic entropy, discrete semantic entropy and naive entropy, we consider two strong baselines.

Embedding regression is a supervised baseline inspired by the *P*(IK) method^24^. In that paper, the authors fine-tune their proprietary LLM on a dataset of questions to predict whether the model would have been correct. This requires access to a dataset of ground-truth answers to the questions. Rather than fine-tuning the entire LLM in this way, we simply take the final hidden units and train a logistic regression classifier to make the same prediction. By contrast to their method, this is much simpler because it does not require fine-tuning the entire language model, as well as being more reproducible because the solution to the logistic regression optimization problem is not as seed-dependent as the fine-tuning procedure. As expected, this supervised approach performs well in-distribution but fails when the distribution of questions is different from that on which the classifier is trained.

The second baseline we consider is the *P*(True) method^24^, in which the model first samples *M* answers (identically to our semantic entropy approach) and then is prompted with the list of all answers generated followed by the highest probability answer and a question whether this answer is “(a) True” or “(b) False”. The confidence score is then taken to be the probability with which the LLM responds with ‘a’ to the multiple-choice question. The performance of this method is boosted with a few-shot prompt, in which up to 20 examples from the training set are randomly chosen, filled in as above, but then provided with the actual ground truth of whether the proposed answer was true or false. In this way, the method can be considered as supervised ‘in-context’ because it makes use of some ground-truth training labels but can be used without retraining the model. Because of context-size constraints, this method cannot fit a full 20 few-shot examples in the context when input questions are long or large numbers of generations are used. As a result, we sometimes have to reduce the number of few-shot examples to suit the context size and we note this in the Supplementary Material.

#### Entailment estimator

Any NLI classification system could be used for our bidirectional entailment clustering algorithm. We consider two different kinds of entailment detector.

One option is to use an instruction-tuned LLM such as LLaMA 2, GPT-3.5 (Turbo 1106) or GPT-4 to predict entailment between generations. We use the following prompt:

We are evaluating answers to the question {question}

Here are two possible answers:

Possible Answer 1: {text1}

Possible Answer 2: {text2}

Does Possible Answer 1 semantically entail Possible Answer 2? Respond with entailment, contradiction, or neutral.

Alternatively, we consider using a language model trained for entailment prediction, specifically the DeBERTa-large model^56^ fine-tuned on the NLI dataset MNLI^58^. This builds on past work towards paraphrase identification based on embedding similarity^59,60^ and BERT-style models^61,62^. We template more simply, checking if DeBERTa predicts entailment between the concatenation of the question and one answer and the concatenation of the question and another answer. Note that DeBERTa-large is a relatively lightweight model with only 1.5B parameters which is much less powerful than most of the LLMs under study.

In Supplementary Note 2, we carefully evaluate the benefits and drawbacks of these methods for entailment prediction. We settle on using GPT-3.5 with the above prompt, as its entailment predictions agree well with human raters and lead to good confabulation detection performance.

In Supplementary Note 3, we provide a discussion of the computational cost and choosing the number of generations for reliable clustering.

#### Prompting templates

We use a simple generation template for all sentence-length answer datasets:

Answer the following question in a single brief but complete sentence.

Question: {question}

Answer:

#### Metrics and accuracy measurements

We use three main metrics to evaluate our method: AUROC, rejection accuracy and AURAC. Each of these is grounded in an automated factuality estimation measurement relative to the reference answers provided by the datasets that we use.

##### AUROC, rejection accuracy and AURAC

First, we use the AUROC curve, which measures the reliability of a classifier accounting for both precision and recall. The AUROC can be interpreted as the probability that a randomly chosen correct answer has been assigned a higher confidence score than a randomly chosen incorrect answer. For a perfect classifier, this is 1.

Second, we compute the ‘rejection accuracy at *X*%’, which is the question-answering accuracy of the model on the most-confident *X*% of the inputs as identified by the respective uncertainty method. If an uncertainty method works well, predictions on the confident subset should be more accurate than predictions on the excluded subset and the rejection accuracy should increase as we reject more inputs.

To summarize this statistic we compute the AURAC—the total area enclosed by the accuracies at all cut-off percentages *X*%. This should increase towards 1 as given uncertainty method becomes more accurate and better at detecting likely-inaccurate responses but it is more sensitive to the overall accuracy of the model than the AUROC metric.

In Supplementary Note 5, we provide the unaggregated rejection accuracies for sentence-length generations.

##### Assessing accuracy

For the short-phrase-length generation setting presented in Supplementary Note 7, we simply assess the accuracy of the generations by checking if the F1 score of the commonly used SQuAD metric exceeds 0.5. There are limitations to such simple scoring rules^63^ but this method is widely used in practice and its error is comparatively small on these standard datasets.

For our default scenario, the longer sentence-length generations, this measure fails, as the overlap between the short reference answer and our long model answer is invariably too small. For sentence-length generations, we therefore automatically determine whether an answer to the question is correct or incorrect by using GPT-4 to compare the given answer to the reference answer. We use the template:

We are assessing the quality of answers to the following question: {question}

The expected answer is: {reference answer}

The proposed answer is: {predicted answer}

Within the context of the question, does the proposed answer mean the same as the expected answer? Respond only with yes or no.

We make a small modification for datasets with several reference answers: line two becomes “The following are expected answers to this question:” and the final line asks “does the proposed answer mean the same as any of the expected answers?”.

In Supplementary Note 6, we check the quality of our automated ground-truth evaluations against human judgement by hand. We find that GPT-4 gives the best results for determining model accuracy and thus use it in all our sentence-length experiments.

### Detecting confabulations in biographies

In this section we describe the application of semantic entropy to confabulation detection in longer model generations, specifically paragraph-length biographies.

We introduce a biography-generation dataset—FactualBio—available alongside this paper. FactualBio is a collection of biographies of individuals who are notable enough to have Wikipedia pages but not notable enough to have large amounts of detailed coverage, generated by GPT-4 (v.0613). To generate the dataset, we randomly sampled 21 individuals from the WikiBio dataset^64^. For each biography, we generated a list of factual claims contained in each biography using GPT-4, with 150 total factual claims (the total number is only coincidentally a round number). For each of these factual claims, we manually determined whether the claim was correct or incorrect. Out of 150 claims, 45 were incorrect. As before, we apply confabulation detection to detect incorrect model predictions, even though there may be model errors which are not confabulations.

#### Prompting and generation

Given a paragraph-length piece of LLM-generated text, we apply the following sequence of steps:

1. Automatically decompose the paragraph into specific factual claims using an LLM (not necessarily the same as the original).
2. For each factual claim, use an LLM to automatically construct *Q* questions which might have produced that claim.
3. For each question, prompt the original LLM to generate *M* answers.
4. For each question, compute the semantic entropy of the answers, including the original factual claim.
5. Average the semantic entropies over the questions to arrive at a score for the original factual claim.

We pursue this slightly indirect way of generating answers because we find that simply resampling each sentence creates variation unrelated to the uncertainty of the model about the factual claim, such as differences in paragraph structure.

We decompose the paragraph into factual claims using the following prompt:

Please list the specific factual propositions included in the answer above. Be complete and do not leave any factual claims out. Provide each claim as a separate sentence in a separate bullet point.

We found that we agreed with the decompositions in all cases in the dataset.

We then generate six questions for each of the facts from the decomposition. We generate these questions by prompting the model twice with the following:

Following this text:

{text so far}

You see the sentence:

{proposition}

Generate a list of three questions, that might have generated the sentence in the context of the preceding original text, as well as their answers. Please do not use specific facts that appear in the follow-up sentence when formulating the question. Make the questions and answers diverse. Avoid yes-no questions. The answers should not be a full sentence and as short as possible, e.g. only a name, place, or thing. Use the format “1. {question} – {answer}”.

These questions are not necessarily well-targeted and the difficulty of this step is the main source of errors in the procedure. We generate three questions with each prompt, as this encourages diversity of the questions, each question targeting a different aspect of the fact. However, we observed that the generated questions will sometimes miss obvious aspects of the fact. Executing the above prompt twice (for a total of six questions) can improve coverage. We also ask for brief answers because the current version of GPT-4 tends to give long, convoluted and highly hedged answers unless explicitly told not to.

Then, for each question, we generate three new answers using the following prompt:

We are writing an answer to the question “{user question}”. So far we have written:

{text so far}

The next sentence should be the answer to the following question:

{question}

Please answer this question. Do not answer in a full sentence. Answer with as few words as possible, e.g. only a name, place, or thing.

We then compute the semantic entropy over these answers plus the original factual claim. Including the original fact ensures that the estimator remains grounded in the original claim and helps detect situations in which the question has been interpreted completely differently from the original context. We make a small modification to handle the fact that GPT-4 generations often include refusals to answer questions. These refusals were not something we commonly observe in our experiments with LLaMA 2, Falcon or Mistral models. If more than half of the answers include one of the strings ‘not available’, ‘not provided’, ‘unknown’ or ‘unclear’ then we treat the semantic uncertainty as maximal.

We then average the semantic entropies for each question corresponding to the factual claim to get an entropy for this factual claim.

Despite the extra assumptions and complexity, we find that this method greatly outperforms the baselines.

#### Entailment estimator

To compute semantic entailment between the original claim and regenerated answers, we rely on the DeBERTa entailment prediction model as we find empirically that DeBERTa predictions result in higher train-set AUROC than other methods. Because DeBERTa has slightly lower recall than GPT-3.5/4, we use a modified set-up for which we say the answers mean the same as each other if at least one of them entails the other and neither is seen to contradict the other—a kind of ‘non-defeating’ bidirectional entailment check rather than true bidirectional entailment. The good performance of DeBERTa in this scenario is not surprising as both factual claims and regenerated answers are relatively short. We refer to Supplementary Notes 2 and 3 for ablations and experiments regarding our choice of entailment estimator for paragraph-length generations.

#### Baselines

We implement two baselines. First, we implement a variant of the *P*(True) method, which is adapted to the new setting. For each factoid, we generate a question with answers in the same way as for semantic entropy. We then use the following prompt:

Question: {question}

Here are some brainstormed ideas:

{list of regenerated answers}

Possible answer: {original answer}

Is the possible answer true? Respond with “yes” or “no”.

As we cannot access the probabilities GPT-4 assigns to predicting ‘yes’ and ‘no’ as the next token, we approximate this using Monte Carlo samples. Concretely, we execute the above prompt ten times (at temperature 1) and then take the fraction of answers which was ‘yes’ as our unbiased Monte Carlo estimate of the token probability GPT-4 assigns to ‘yes’.

As a second, simpler, baseline we check if the model thinks the answer is true. We simply ask:

Following this text:

{text so far}

You see this statement:

{proposition}

Is it likely that the statement is true? Respond with ‘yes’ or ‘no’.

It is interesting that this method ought to perform very well if we think that the model has good ‘self-knowledge’ (that is, if “models mostly know what they don’t know”^24^) but in fact semantic entropy is much better at detecting confabulations.

## Online content

Any methods, additional references, Nature Portfolio reporting summaries, source data, extended data, supplementary information, acknowledgements, peer review information; details of author contributions and competing interests; and statements of data and code availability are available at 10.1038/s41586-024-07421-0.

### Supplementary information

Supplementary Information Supplementary Notes 1–7, Figs. 1–10, Tables 1–4 and references. Includes, worked example for semantic entropy calculation, discussion of limitations and computational cost of entailment clustering, ablation of entailment prediction and clustering methods, discussion of automated accuracy assessment, unaggregated results for sentence-length generations and further results for short-phrase generations.

## Data Availability

The data used for the short-phrase and sentence-length generations are publicly available and the released code details how to access it. We release a public version of the FactualBio dataset as part of the code base for reproducing the paragraph-length experiments.

## Code availability

We release all code used to produce the main experiments. The code for short-phrase and sentence-length experiments can be found at github.com/jlko/semantic_uncertainty and 10.5281/zenodo.10964366 (ref. ^65^). The code for paragraph-length experiments can be found at github.com/jlko/long_hallucinations and 10.5281/zenodo.10964366 (ref. ^65^).
