# Supplementary material for: Detecting hallucinations in large language models using semantic entropy
Source: Nature. 2024 Jun 19;630(8017):625–30. doi: 10.1038/s41586-024-07421-0 (PMC11186750; doi:10.1038/s41586-024-07421-0)
Supplement: Supplementary file 1 — Supplementary Notes 1–7, Figs. 1–10, Tables 1–4 and references. Includes, worked example for semantic entropy calculation, discussion of limitations and computational cost of entailment clustering, ablation of entailment prediction and clustering methods, discussion of automated accuracy assessment, unaggregated results for sentence-length generations and further results for short-phrase generations. [file 41586_2024_7421_MOESM1_ESM.pdf]

nature portfolio

https://doi.org/10.1038/s41586-024-07421-0

Supplementary information

Detecting hallucinations in large language models using semantic entropy

In the format provided by the authors and unedited

Supplementary Material

Note 1: Worked Example of Semantic Entropy Calculation

This note provides a worked example of the calculation of semantic entropy. As a worked example, suppose that we have asked "Where is the Eiffel Tower?". The model generates five answers with the length-normalised sequence log-probabilities $\frac{1}{N}\prod_i^N p(\mathbf{s}_i \mid \mathbf{s}_i)$ (given in the first column). In this hypothetical example, we happened to sample the literal string "Paris." twice, because we are just randomly sampling from the language model which assigns the string high probability. But we also found a different string that was equivalent to it, as well as some wrong answers. Note that in the case of model APIs which do not report the log-probabilities (such as GPT-4 at time of writing) we will not have the final number, just the text. To compute the semantic entropy, we cluster these generations into clusters that can be considered to mean the same thing. We also add up the probabilities associated

		Naive entropy			Semantic entropy			
Generation	p	$p(\mathbf{s}_i)$	$\log[p(\mathbf{s}_i)]$	$p(\mathbf{s}_i)\log[p(\mathbf{s}_i)]$	$\sum_{\mathbf{s}_i \in C_j} p(\mathbf{s}_i)$	$p(C_j)$	$\log[p(C_j)]$	$p(C_j)\log[p(C_j)]$
"Paris."	0.20	0.33	−0.48	−0.16	0.55	0.90	−0.04	−0.04
"Paris."	0.20	0.33	−0.48	−0.16	-	-	-	
"It's Paris."	0.15	0.25	−0.61	−0.15	-	-	-	
"Rome."	0.05	0.08	−1.09	−0.09	0.05	0.08	−1.09	−0.09
"New York."	0.01	0.02	−1.79	−0.03	0.01	0.02	-1.79	−0.03
Sum	0.61	1.00	−4.45	**−0.59**	0.61	1.00	−2.92	**−0.16**

Supplementary Table 1: **Worked example of Semantic Entropy Calculation.** The raw token sequence probabilities for each generation, p, are in the first column. Note that they do not sum to one because they are the probabilities associated with each actually sampled outcome, and if we sample many generations their sum will exceed one. To calculate the naive entropy of the output distribution, in the second column we compute an estimator of the normalised probability for each generated sequence, $p(\mathbf{s}_i)$, by dividing each probability by the sum of the first column. These now do sum to one (up to a rounding error). One way to estimate the naive entropy would then be to multiply each log-probability by the probability, sum them (and multiply by -1, not shown). For the semantic entropy, we instead look at the probabilities summed up within each meaning-cluster. We compute log-probabilities in the same way, and then compute the entropy of the resulting distribution. The resulting entropy is much lower, because several generations meant the same thing as each other (final column). All values to two decimal places.

with each one. This value is reported in the fifth column (the first under the heading "Semantic entropy"). To compute the semantic entropy we compute the negative sum of the expectation of the log-probabilities (Eq. (5)). That is, we get the result $0.16 = -0.9\log(0.9) - 0.08\log(0.08) - 0.02\log(0.02)$.

For the "discrete" variant of semantic entropy we effectively treat the probability of sampling each of the generations as uniform, by using it as an empirical distribution that approximates the underlying distribution. This means that for the "Paris." cluster we get a weight of 0.6=0.2+0.2+0.2 and a weight of 0.2 for the other two clusters. That is, the discrete semantic entropy here is $0.41 = -0.6\log(0.6) - 0.2\log(0.2) - 0.2\log(0.2)$.

Although these two methods produce different absolute results, we find that in practice they tend to agree fairly well on relative ordering, which is what is used in practice to classify confabulations. As a result, the cluster approximation of semantic entropy is a fairly good alternative in cases where the log-probabilities are not disclosed.

Note 2: Choosing an Entailment Estimator

Sentence-length Generations

We confirm that the bi-directional entailment classifier works as expected. Prior work has show that in some settings NLI methods can systematically fail[78], so we seek to ascertain whether these failures substantially affect typical question-answering. Two raters manually labeled 100 pairs of sentence-length generations from LLaMA 2 Chat 70B for three of our datasets for entailment, recording whether they believed that sentence A entailed sentence B. They rated each entailment as: `entailment`, `neutral`, `contradiction`. For the purpose of measuring agreement we combine the neutral and contradiction ratings, because our method is searching for positive entailment. We found that the human raters agreed with each other (87%) at roughly the same rate that they on average agreed with GPT-4 (87%) while they agreed with GPT-3.5 only

	DeBERTa	LLaMA 2 Chat 70B	GPT-3.5	GPT-4	Human A	Human B
Human A	0.81	0.78	0.83	0.89	-	0.87
Human B	0.78	0.80	0.84	0.85	0.87	-
Human Average	0.80	0.79	0.83	0.87	-	-

Supplementary Table 2: **Manual entailment evaluation.** Inter-rater agreement on entailment classification for pairs of sentence-length answers produced by LLaMA 2 Chat 70B to 100 questions from each of SQuAD, TriviaQA, and BioASQ (600 answers in total). On average, the human raters agreed with each other to approximately the same extent that they agreed with GPT-4, while GPT-3.5 was only slightly less predictive of human-assessed entailment.

slightly less on average (83%). As a result, because GPT-3.5 is more than an order of magnitude cheaper, we use GPT-3.5 for all entailment calculations for sentence-length generations on SQuAD, TriviaQA, BioASQ, SVAMP, and NQ Open. Presumably our method would perform better with a more expensive entailment estimator. Supplementary table 2 shows the detailed agreement results between the human raters and the entailment estimation.

In addition to validating human agreement with the entailment models, we also investigate the performance of semantic entropy with different entailment strategies. In supplementary table 3, we report AUROC values of semantic entropy with various entailment models for predictions from a LLaMA 2 Chat 70B model on TriviaQA, SQuAD, and BioASQ. The LLaMA 2 Chat 70B model performs worst, followed by the purpose-built DeBERTa model and GPT models, where this time version 3.5 slightly outperforms version 4 on average. For these experiments, unlike our main results, we used only 8 generations (normally 10) to estimate the entropy and measured accuracy relative to the reference answer using LLaMA 2 Chat 70B (normally GPT-4, see section on 'Assessing Accuracy').

	DeBERTa	LLaMA 2 Chat 70B	GPT-3.5	GPT-4
TriviaQA	0.83	0.70	0.85	0.83
SQuAD	0.76	0.71	0.77	0.80
BioASQ	0.75	0.73	0.87	0.79
Average	0.78	0.71	0.83	0.80

Supplementary Table 3: **Entailment Method Ablation.** AUROC values for semantic entropy when using different models to compute entailment for sentence-length generations from LLaMA 2 Chat 70B. Semantic entropy performs better when prompted GPT models predict entailment rather than a purpose-built DeBERTa model.

Paragraph-length Generations

In supplementary figure 1, we report our experiments for several entailment prediction variants for paragraph-length generations, in addition to our default non-defating bi-directional DeBERTa method. We also experimented with several entailment variants: "GPT-4 two-way" asks GPT-4 to evaluate whether the sentences mean the same thing directly ("Do the following two possible answers to the subquestion mean the same thing?" instead of "Does Possible Answer 1 semantically entail…"); "GPT-4 'equivalent"' instead asks "Are the following two possible answers to the subquestion semantically equivalent?"; while "GPT-4 simultaneous 'equivalent' " provides all of the possible answers and asks "Are the following answers equivalent?". All of these methods were substantially worse.

Note 3: Limitations to Clustering by Entailment

In idealized examples, it is clear when two sentences do or do not mean the same thing as each other. In practice, it can sometimes be that sentence A seems to mean the same as B, and B the same as C, but A and C don't seem to mean the same thing. That is, because semantic equivalence is fuzzy it does not always intuitively behave transitively, meaning that the assumptions behind our equivalence classes do not hold in practice.

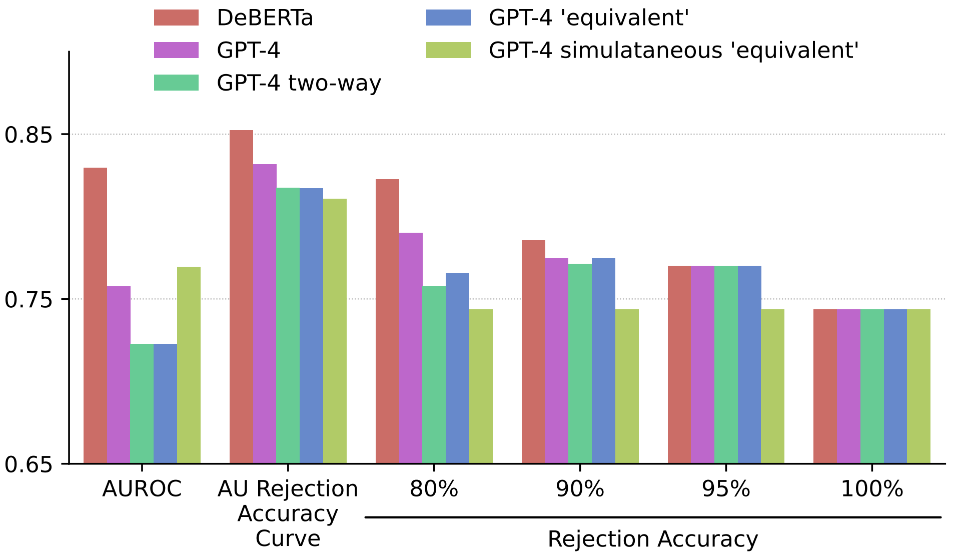

Supplementary Figure 1: **Entailment method choice for paragraph biographies.** Implementing the non-defeating bi-directional entailment with DeBERTa provided the best empirical results for paragraph biography confabulation detection.

Similarly, there are cases where bi-directional entailment does *not* mean that two sentences mean the same thing. For example, "John drove his car to the store." and "John went to the store in his car." generally imply each other and would be marked as "entailment" by most classifiers, and this reflects the fact that they mean more-or-less the same thing. But, for example, this depends somewhat on the context and various aspects of implicature[76]. For example, if we have other reasons to think that John might have been the owner of the car but a passenger, rather than the driver, then we might judge the two sentences to not mean the same thing as each other. As an alternative example of a failure of bi-directional entailment to correspond with semantic equivalence, sentences with scalar adverbs such as "Paris might be in France" and "Paris might not be in France" can entail each other while meaning something quite different[77].

For questions whose answers are straightforward, relatively objective, factual, and not vague these problems may not be significant. In particular, we did not observe any of these problems arising in manual inspection of outputs during any of our experiments. Nevertheless, for subtle situations and applications we would encourage practitioners to check these assumptions.

Note 4: Computational Cost and Choosing the Number of Generations

The bi-directional equivalence algorithm is combinatorially complex in M, the number of samples generated, as it requires $\binom{M}{2}$-many comparisons in the worst-case. In practice, however, the computational cost is small compared to the cost of generating sequences.

First, M does not necessarily need to be very large. We show how the confabulation-detection performance (measured by AUROC) changes with M for sentence-length generations in supplementary figure 2 and for FactualBio paragraphs in supplementary figure 3. For sentence-length generations, after roughly $M = 5$ there are diminishing returns, although going up to $M = 10$ can still help. In this paper, we use $M = 10$ for sentence-length generations as well as short-phrase generations. For this ablation, we produce generations using LLaMA 2 Chat 70B but several experimental characteristics differ from those of our main results. We check entailment using GPT-4 (standardly GPT-3.5), measure accuracy using LLaMA 2 Chat 70B (standardly GPT-4), and use 8 generations to estimate entropy (standardly 10). For paragraph-length biographies, we find that four total factoids (three new generations plus the original factoid) seems optimal (see supplementary figure 3). Unlike the standard setting, more generations is not strictly better, because it decreases the relative weight on the original factoid which increases the risk of a badly posed question that generates irrelevant answers.

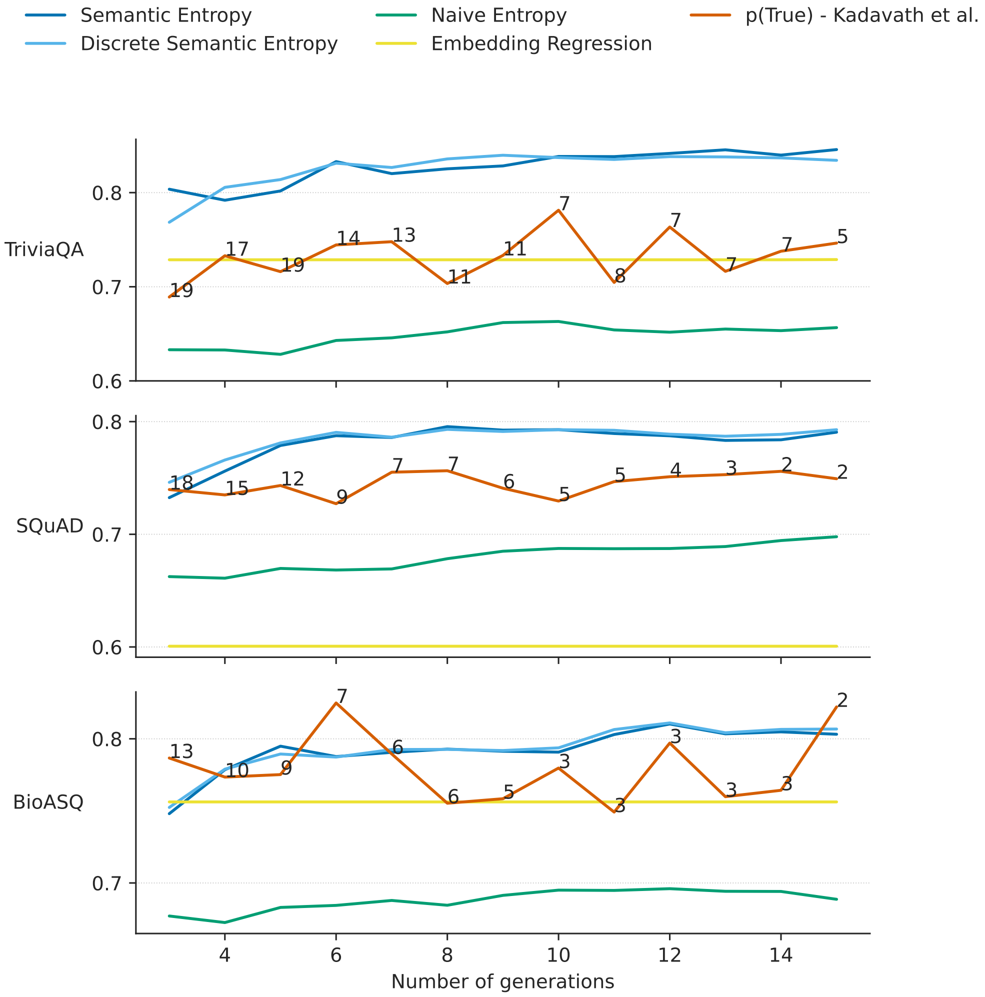

Supplementary Figure 2: **Number of sentence-length generations used for entropy.** We find diminishing returns to increasing the number of generations sampled for the semantic entropy estimation, but select 10 as a reasonable number for results in this paper. The numbers annotating $p(\text{True})$ illustrate the number of few-shot examples we were able to include without exceeding the maximal input size for each dataset and number of generations.

Second, when using the DeBERTa-large model, it is so much smaller than the main language model, each pair comparison is much faster than generating even one token from the main model. Using GPT-3.5 to do clustering is considerably more expensive than DeBERTa.

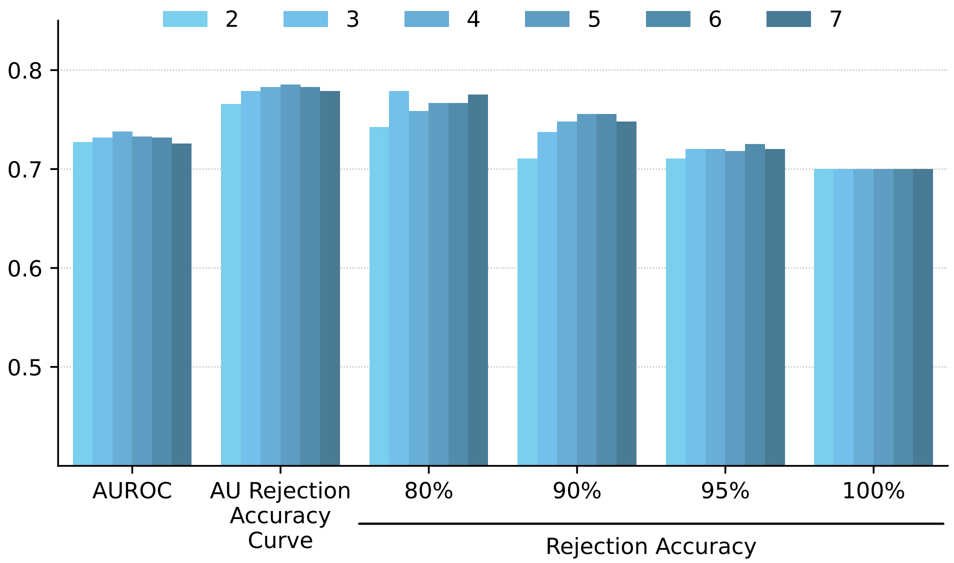

Supplementary Figure 3: **Number of factoid generations used in paragraph-length biographies.** We find that confabulation-detection performance is not very sensitive to the number of generations, but that four total factoids per question (including the original one) results in competitive performance.

Third, because semantic equivalence is transitive we only need to compare one member of each equivalence class to the remaining sequences (see algorithm in Extended Data Figure 1). The number of semantic clusters in our tasks is empirically often quite low which means that far fewer than the worst-case number of comparisons are actually needed in practice. In supplementary figure 4, we show some empirical numbers of clusters for several key datasets.

Fourth, because the LLM often generates identical sequences in practice, we can cache entailments. For example, if the LLM's three generations in response to a question are "Paris.", "It's in Paris.", and "Paris." we can do a (very computationally cheap) *string level* comparison of the final "Paris." to the previous generations and, on finding that it is the identical text to the earlier string, we can use the previously calculated entailments. We find that in practice this reduces the computational costs

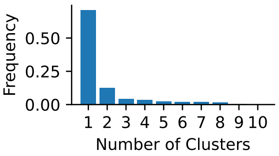

(a) Sentence—TriviaQA.

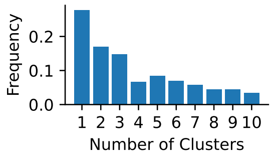

(b) Sentence—SQuAD.

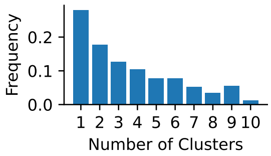

(c) Sentence—BioASQ.

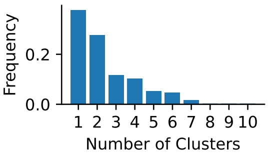

(d) Sentence—SVAMP.

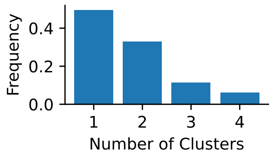

(e) Paragraph—4 facts

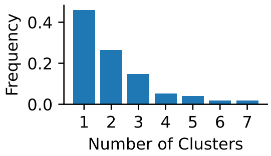

(f) Paragraph—7 facts

Supplementary Figure 4: In many cases, relatively few clusters are found, which can improve computational efficiency. The easiest dataset (TriviaQA) generally has fewest clusters because the answers are confident. In our results for the paragraph-length task, we use 4-factoids per question as shown in (supplementary figure 4e), but increasing the number of generations does not greatly increase the number of clusters (supplementary figure 4f). All sentence-length-generation plots are for LLaMA 2 Chat 70B while the paragraph-generation plots are GPT-4.

by 51.4% for TriviaQA, 12.3% for BioASQ, and 18.0% for SQuAD (with the size of the improvement caused by the proportion of identical answers produced by the model for those datasets).

Note 5: Further Details for Sentence-Length Generations.

Here, we provide an unaggregated view of the sentence-length AUROCs which form Figure 2. Individual datasets and models follow a very similar pattern to the average, as shown in supplementary figure 5

We also provide a more detailed view of the rejection accuracies at proportions of answers retained for sentence-length generations in supplementary figure 6.

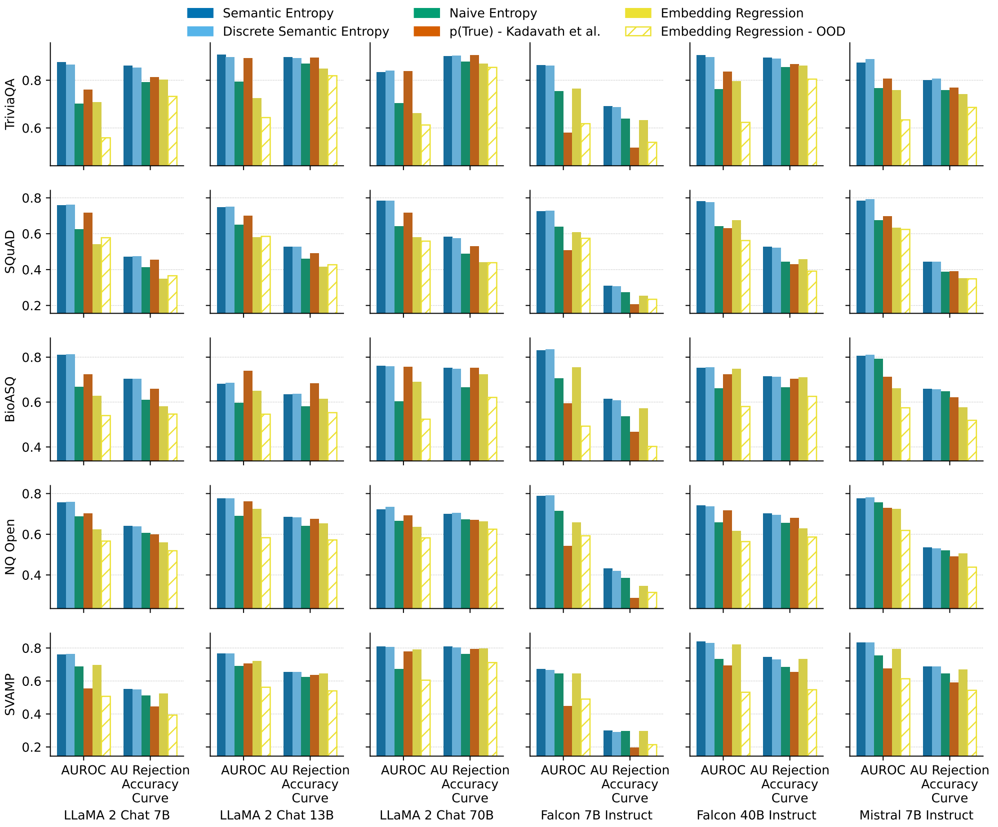

Supplementary Figure 5: **Sentence-length confabulation detection—full AUROC**. An unaggregated view of the AUROCs shown in Figure 2.

Note 6: Assessing Model Accuracy

We check the quality of our automated ground-truth evaluation (using GPT-4 to compare the model generation with the reference answer) against human judgement by hand on sentence-length answers produced by LLaMA 2 Chat 70B responding to 100 questions from TriviaQA, SQuAD, and BioASQ. In each case, we check whether a generated answer matches the reference answer. Even if doing this reveals that the reference answer is wrong, which sometimes happens, we are interested in knowing whether humans and the automatic methods agree on the match, not on whether they know the actual correct answer. Supplementary table 4 shows the two human raters

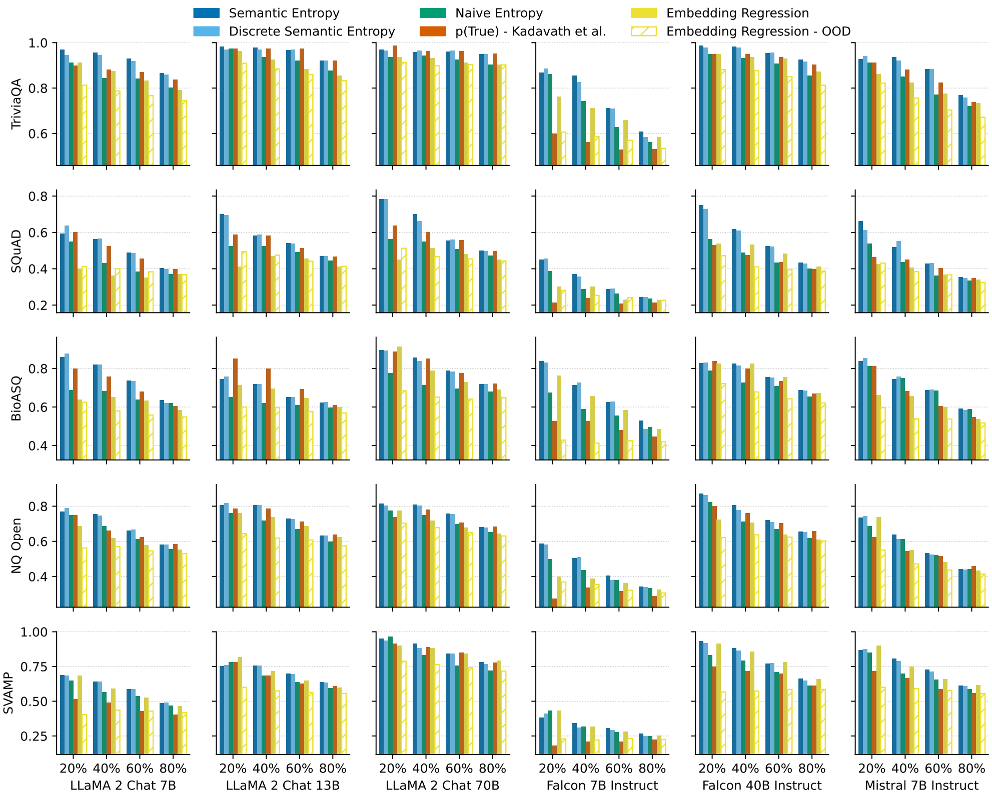

Supplementary Figure 6: **Sentence-length confabulation detection—rejection accuracy**. A more detailed view of the rejection accuracies of the figures provides further elaboration of the findings in Figure 2.

agreed with each other at roughly the same rate (92%) as they agreed with GPT-4 on average (93%). While GPT-3.5 is only slightly worse, in order to get the best ground-truth estimation feasible, we use GPT-4 to compare the generated answer with the reference answers provided in the dataset for results in this paper.

Note 7: Results for Short-Phrase Generation

In the main text, we provide results for sentence-length and paragraph-length generation. Here, we show results for a "short-phrase" scenario where we prompt the model to "Answer the following question as briefly as possible". Additionally, we here

	F1	LLaMA 2 Chat 70B	GPT-3.5	GPT-4	Human A	Human B
Human A	0.48	0.92	0.88	0.92	-	0.92
Human B	0.47	0.93	0.90	0.93	0.92	-
Human Average	0.47	0.92	0.89	0.93	-	-

Supplementary Table 4: **Ground truth evaluation.** We evaluate different automatic accuracy measures against human evaluation on sentence-length answers produced by LLaMA 2 Chat 70B on 100 randomly chosen questions from TriviaQA, SQuAD, and BioASQ. We find that GPT-4 agrees with both human raters at roughly the same level as they agree with each other.

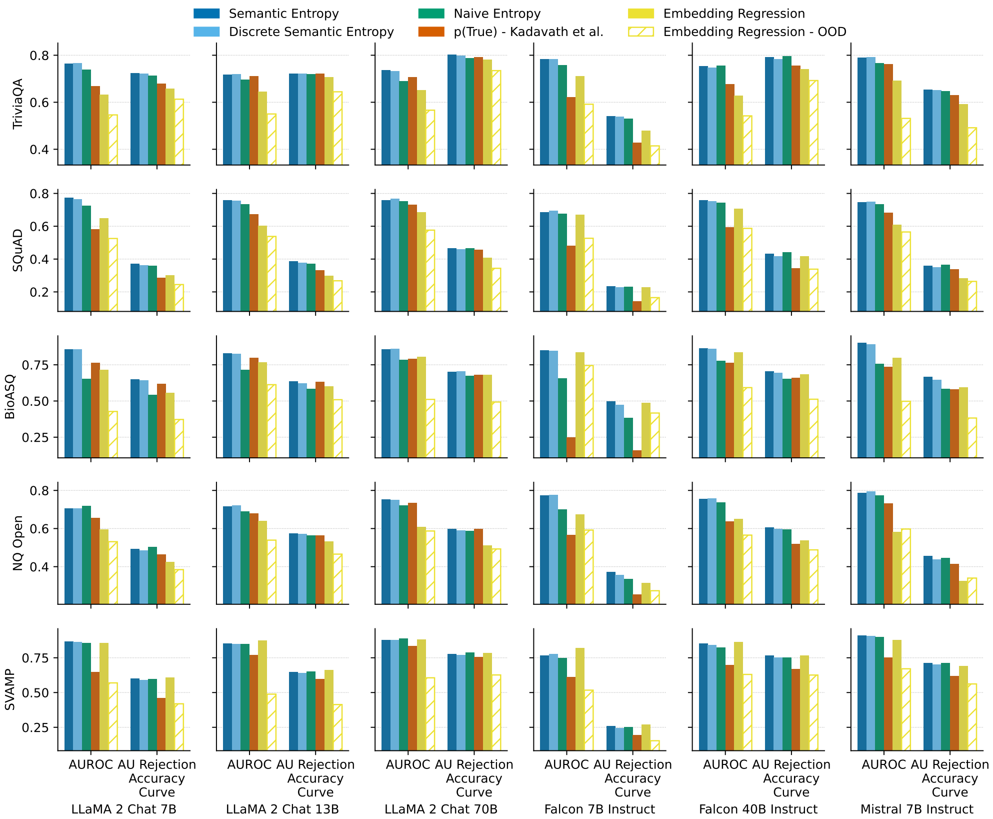

Supplementary Figure 7: **Short-Phrase confabulation detection**. With a prompt that encourages short generations, we achieve a lower average answer length (15.9±21.8 characters compared to 95.6±69.6 for sentence-length generations). Semantic entropy still works well compared to p(True) and embedding regression, although its advantage over naive entropy is smaller to due to the lower syntactic variability of the shorter generations.

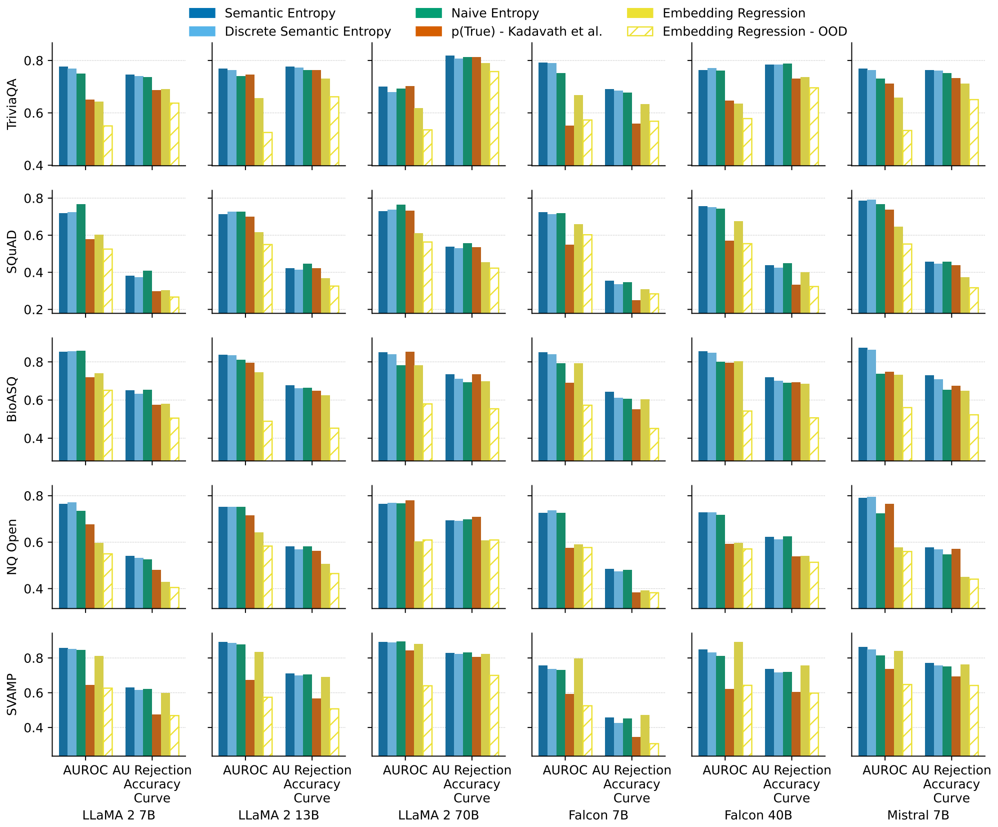

Supplementary Figure 8: **Short-Phrase confabulation detection—Non-instruction-tuned models**. Models that have not been instruction-tuned have slightly different output-distribution characteristics to instruction-tuned models. However, we find that both classes of models show broadly similar results and that semantic entropy continues to outperform baselines in this setting.

employ five few-shot QA demonstrations before the main question, all following the template introduced above, which further encourages the LLM to predict with brevity as the reference answers are usually very short for our selection of datasets. The short-phrase scenario is less practically relevant, as users commonly interact with longer LLM generations, although some settings value brevity and directness.

For short-phrase generations we use the DeBERTa entailment classifier method to check for strict bi-directional entailment, because this is cheaper and well-suited to short phrases. In addition to the instruction-tuned LLaMA, Falcon, and Mistral

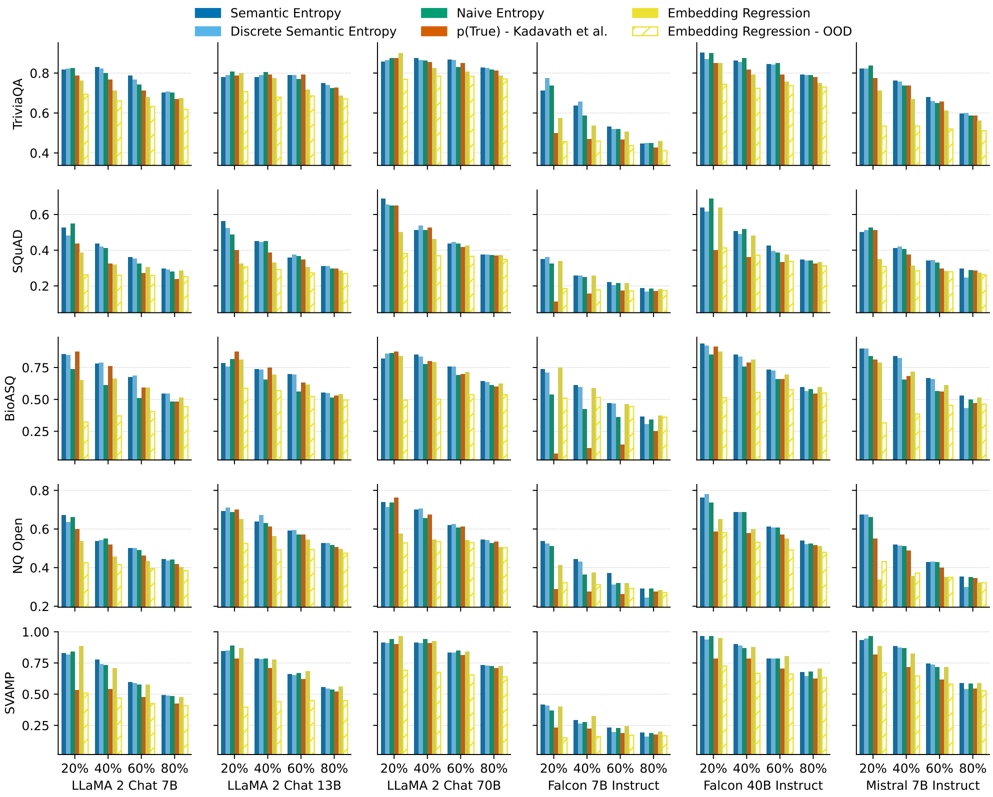

Supplementary Figure 9: **Short-Phrase confabulation detection—rejection accuracy**. A more detailed view of the rejection accuracies of the figures provides further elaboration of the findings in supplementary figure 7.

models we use in the sentence-length experiments, we additionally report results on the non-instruction-tuned LLaMA, Falcon, and Mistral models (which are not effective when applied to the sentence-length generation setting).

In supplementary figure 7, we show that results in this setting are broadly similar to those in the longer setting, with semantic entropy improving over $p(\text{True})$, embedding regression baseline, and naive entropy. In supplementary figure 8 we show this also holds for non-instruction-tuned models. Although non-instruction-tuned models have

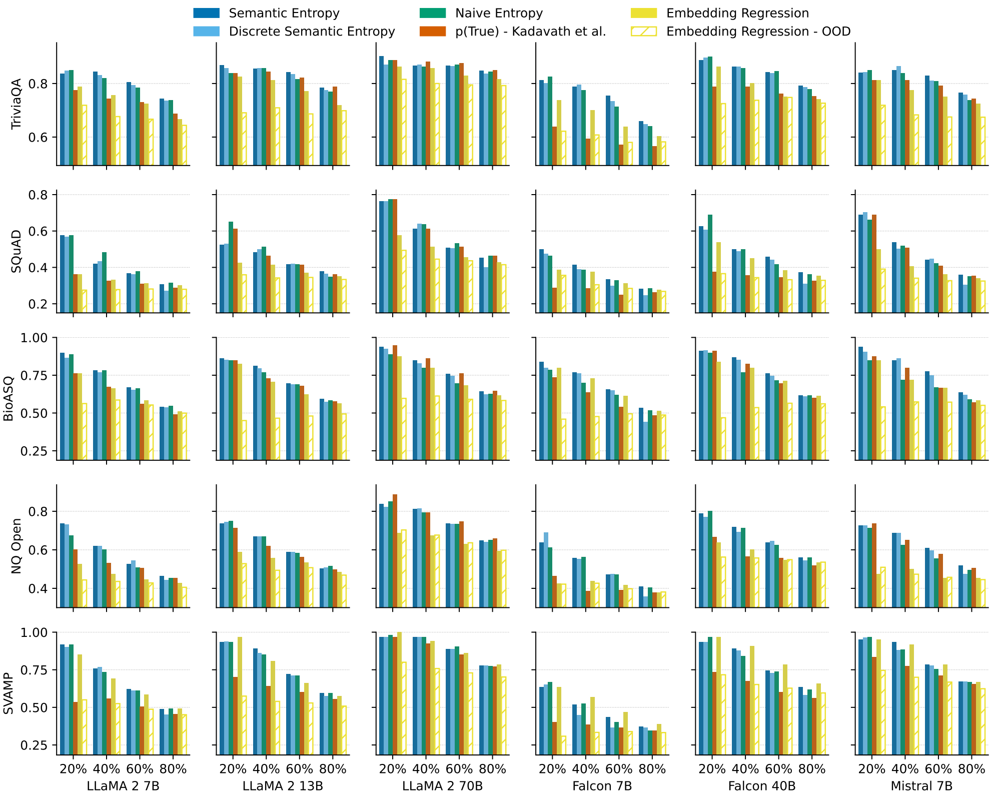

Supplementary Figure 10: **Short-Phrase confabulation detection—rejection accuracy for non-instruction tuned models**. A more detailed view of the rejection accuracies of the figures provides further elaboration of the findings in supplementary figure 7.

slightly different distributional characteristics—tending to have better-calibrated output probabilities—we find that semantic entropy continues to outperform the baselines in this setting.

Averaged across the 60 combinations of tasks and models we study for the short-phrase setting, semantic entropy and discrete semantic entropy achieve the best mean AUROC (see below) values of 0.792 and 0.790 while naive entropy 0.760, $p(\text{True})$ 0.683, and the embedding regression baseline 0.708 lag behind it. Notably, semantic entropy continues to improve over $p(\text{True})$ and the embedding regression baseline by about 0.10 AUROC. However, naive entropy performs better than for longer generations, although

semantic entropy does still improve over it by 0.03 on average (and by significantly more for individual datasets and models). This is because longer answers exhibit more of the syntactic variation that causes naive entropy to fail, and requiring answers to be as short as possible reduces the opportunity for variation.

In supplementary figure 9 we provide a more detailed view of the rejection accuracies at different proportions of answers retained. Lastly, supplementary figure 10 shows rejection accuracies for non-instruction tuned models.

References

[76] Herbert Paul Grice. *Studies in the Way of Words*. Harvard University Press, Cambridge, 1989.

[77] Isabelle Lorge and Janet B. Pierrehumbert. Not wacky vs. definitely wacky: A study of scalar adverbs in pretrained language models. *BlackBoxNLP Workshop at EMNLP*, 2023.

[78] Nick McKenna, Tianyi Li, Liang Cheng, Mohammad Javad Hosseini, Mark Johnson, and Mark Steedman. Sources of hallucination by large language models on inference tasks. In *Findings of EMNLP*, 2023.
